# Giant non-functioning adrenocortical carcinoma: A rare childhood tumor

**DOI:** 10.4103/0971-5851.71659

**Published:** 2010

**Authors:** Viral V. Patel, Diva S. Shah, Chandra R. Raychaudhari, Keyuri B. Patel

**Affiliations:** *Department of Radiodiagnosis, PramukhSwami Medical College and Shree Krishna Hospital, Karamsad, Gujarat, India*

**Keywords:** *Adrenocortical carcinoma*, *adrenocortical tumor*, *nonfunctioning*

## Abstract

Adrenocortical carcinoma (ACC) is a rare malignancy, especially in children. The overall incidence is approximately 2 cases per million per year.[1] In children, the incidence is 0.3 cases per million per year, except in southern Brazil where the incidence is 3.4–4.2 cases per million per year.[2] We describe a giant nonfunctioning metastasized ACC in a 6-year-old girl who presented with a history of increasing abdominal girth incidentally noticed by her mother since 1 week. Ultrasound abdomen showed a large right suprarenal tumor with calcifications and necrosis. Empty left renal fossa and compensatory enlarged right kidney were seen. Computed tomography (CT) scan revealed a large heterogenously enhancing right suprarenal mass with calcification and necrosis with pulmonary metastasis. Histopathology report from the right suprarenal mass revealed an ACC. With a stage IV disease, the patient died after 2 months from diagnosis.

## INTRODUCTION

Adrenocortical carcinoma (ACC) is an unusual, and a highly malignant childhood tumor with grave prognosis. It accounts for 0.002% of childhood malignancies, with most of the tumors being functional in children.[3] Nonfunctioning adrenocortical tumors are exteremly rare in children.[3] There are many case reports for functional ACC, but very few reports are available for non-functioning ACC in children owing to its rarity.[4,5]

The tumor has bimodal age distribution, presenting in children under 6 years, and in adults 30-40 years old.[6] Girls are more frequently affected than boys.[6] Functioning ACC usually draws clinical attention for many hormonal syndromes, viz., virilization, cushing’s syndrome, cons syndrome and feminization.

Nonfunctioning adrenal tumors remain a diagnostic challenge in early diagnosis and successful management as there are no early signs and symptoms. In a majority of cases, the tumor has either invaded adjacent organ or already metastasized to distant organ at the time of initial diagnosis. In most of the cases, it is mistaken for neuroblastoma which is the commonest intra-abdominal childhood tumor.[3] Very rare incidence and unusual mode of presentation in childhood in our patient with a single functioning kidney prompted us to make a case report with its review of literature.

## CASE REPORT

A 5-year-old girl presented with complains of abdominal swelling and low grade fever since few days. No relevant past history of bowel or urinary complains was present. There was also no significant family history of cancer. Physical examination revealed distended abdomen with a palpable lump in the right hypochondrium. Her temperature was mildly raised, blood pressure was 110/72 mm Hg and rest of the vitals were unremarkable.

Ultrasonography of abdomen revealed a large heterogenous mass of size approximately 11×10.6×9.26 cm in the right suprarenal region, with calcifications, large necrotic and hemorrhagic areas within and indistinct fat planes from the superior pole of right kidney. Left kidney was not seen in left renal fossa or anywhere else in abdomen, suggesting possible congenital absence. No focal lesion was observed in liver.

**Figure 1:**
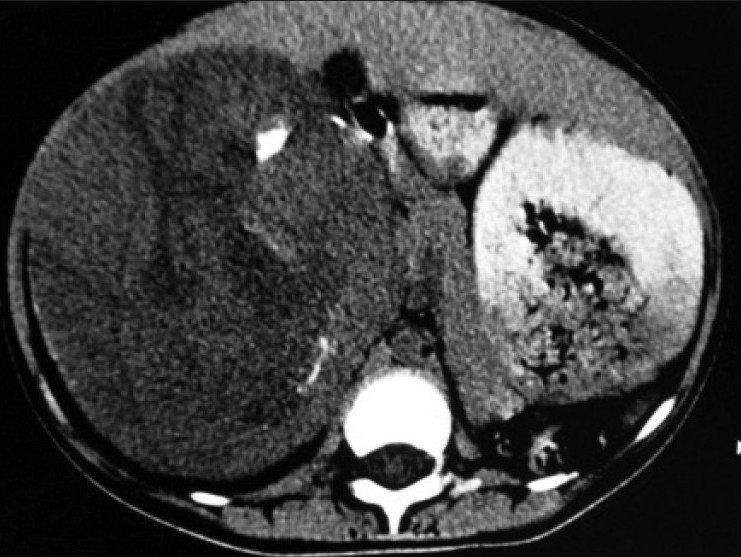
Non-contrast enhanced CT scan revealed large heterogenous mass with few chunks of calcifications

**Figure 2:**
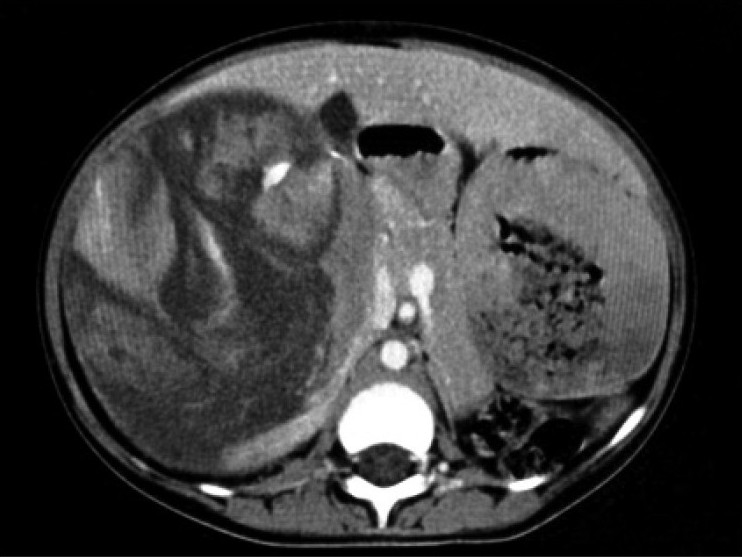
Contrast-enhanced CT scan showing heterogenously enhancing suprarenal lesion with areas of necrosis

**Figure 3:**
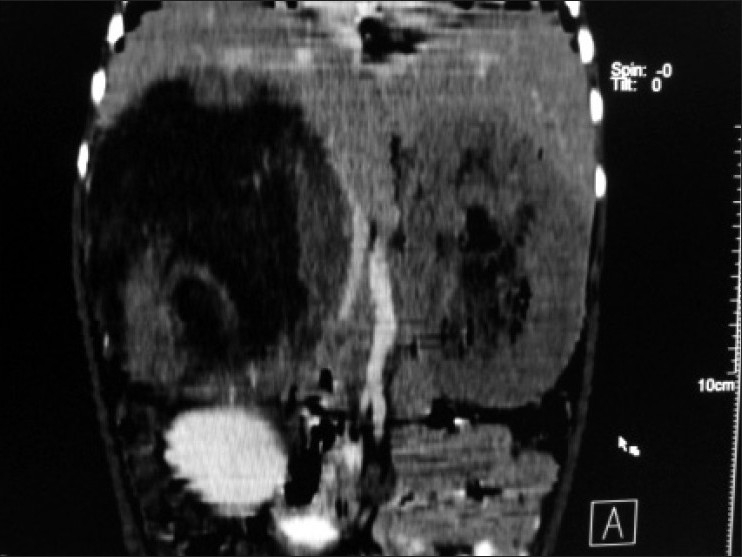
Multiplanner coronal reformation shows right suprarenal mass causing displacement and bowing of inferior vena cava and abutting inferior surface of liver and empty left renal fossa

**Figure 4:**
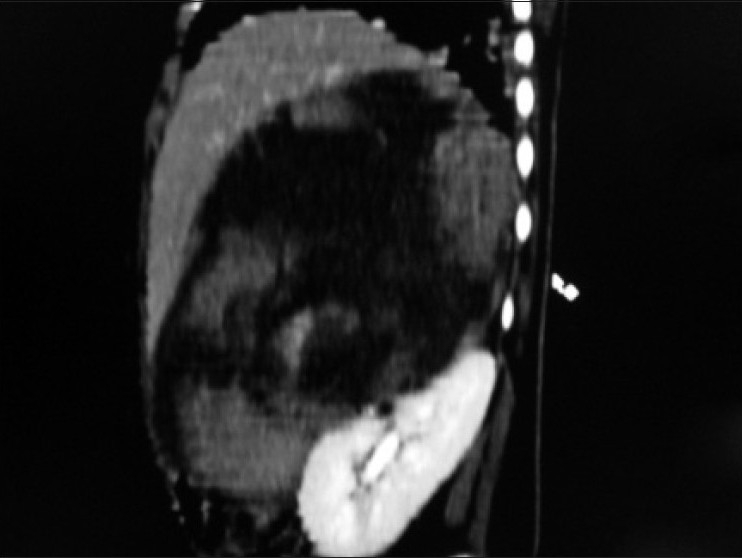
Sagittal multiplanner reformation of right suprarenal mass lesion abutting anterosuperior aspect of right kidney

**Figure 5:**
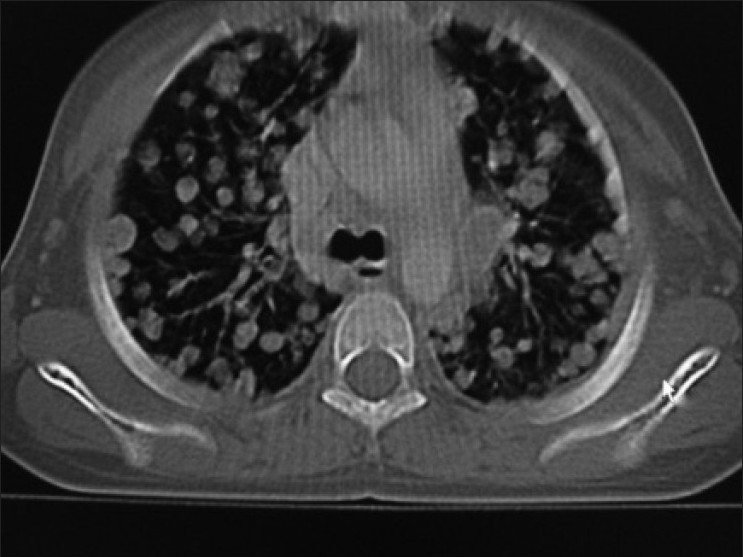
Bilateral randomly distributed variable sized lung parenchymal lesions represent metastasis

Contrast enhanced CT scan of abdomen confirmed the above findings [Figures 1 and 2]. In addition, the lesion was abutting right lobe of the liver causing mass effect and displacing the right branch of portal vein and right hepatic vein [Figures 3 and 4]. Inferiorly, the supero-medial pole of the right kidney had indistinct fat plane with mass lesion. However *the claw sign and organ embedded sign* were negative, suggestive of extra renal origin [Figure 4]. Medially, the lesion was extending in the midline abutting the caudate lobe, causing compression and displacement of inferior vena cava [Figure 3]. It was abutting the renal vessels without any evidence of invasion/thrombosis. Multiple, moderately enhancing round to oval shaped, randomly distributed lesions were observed, involving bilateral lung parenchyma [Figure 5], suggestive of bilateral pulmonary metastatic deposits. There was no evidence of bone marrow/bone metastasis.

**Figure 6:**
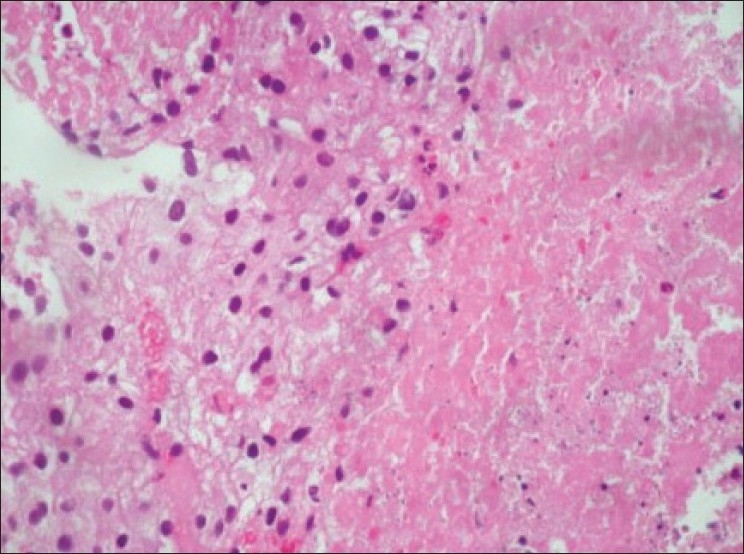
Area of necrosis with adjacent viable cells

**Figure 7:**
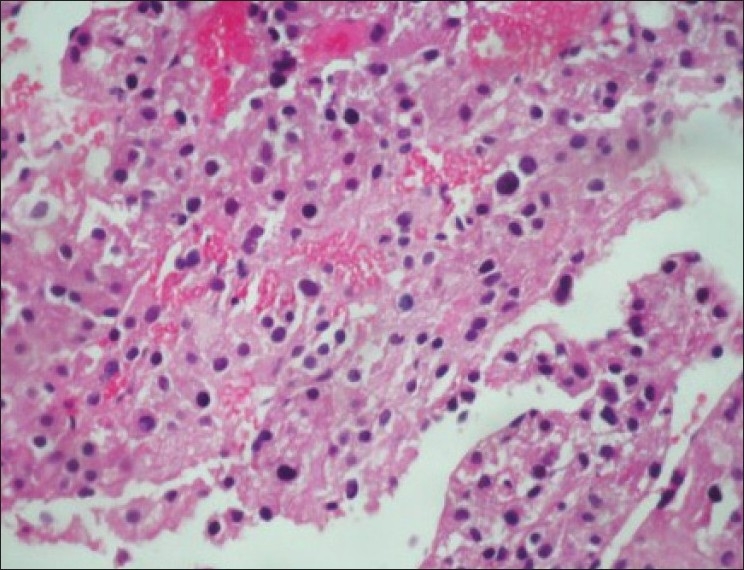
Significant nuclear atypia, hyperchromasia and pleomorphism

Ultrasound guided biopsy revealed ACC. The tru-cut biopsy cores showed areas with patternless sheets of cell interrupted by a fine sinusoidal pattern and broad trabeculae. Wide expanses of necrosis were seen. [Figure 6] The individual cells had predominantly eosinophilic cytoplasm. Less than 25% cells were clear cells. Significant nuclear atypia and hyperchromatism were noted. Infrequent but definitive atypical mitotic figures were seen. [Figure 7] Six out of 9 Weiss criteria were fulfilled. The histologic score as per Van Slooten *et al*. was 21.

Routine laboratory investigations were unremarkable. Although there were no clinically evident signs of virilization or Cushing’s syndrome, plasma cortisol urinary levels of 17-ketosteroids and 17-hydroxycoticosteroid were measured and found to be within normal range.

The patient was grouped as stage IV (according to TNM classification)^1^ and was planned for palliative chemotherapy. As it was an advanced stage disease with metastasis and considering the remote chances of complete cure and the costly chemotherapy drugs which were not affordable by the parents, they decided to start ayurvedic treatment. The patient survived for 2 months after diagnosis.

## DISCUSSION

ACC is an extremely rare tumor. In children, 90% of the adrenal tumors are neuroblastomas (adrenal medulla). Tumors arising from adrenal cortex are rare. Among them, ACC is most common and it accounts for only 6% of adrenal tumors. Adrenal tumors in children can be associated with hemihypertrophy and Beckwith Wiedmann syndrome.[3]

ACC are classified as functional and nonfunctional based on the hormonal syndromes they produce. Functional tumors are common and detected earlier than nonfunctioning tumors due to the production of hormones and associated clinical signs as well as symptoms.[6] Nonfunctioning tumors remain undiagnosed till late and mostly present with a large mass and metastatic disease, as in our case, due to their silent nature.[4,5] Adrenocortical tumors are associated with fever for unknown reasons, as seen in our case.[7]

Primary adrenocortical tumors are large tumors usually measuring more than 5 cm at presentation. The larger the tumor, more is the chance of it being malignant. Because they are large, the organ of origin often is difficult to determine. CT scan plays an important role in characterizing the organ of origin and in defining the extent of the primary as well as assessing the presence of metastatic disease. The common metastatic sites include lung and liver, with bone and bone marrow being less common.[3] Neuroblastoma, which is a more common childhood tumor with similar location, has a tendency to metastasize to bone and bone marrow, though there are case reports showing increasing prevalence of lung metastasis.[8] However, in neuroblastoma, pulmonary metastasis is usually a terminal event where at least one other metastatic site apart from lung is present, which could be bone, bone marrow or liver.[8]

ACC tends to be highly malignant and locally invasive, and potential curative treatment is complete surgical removal. The role of tumor debulking in metastatic ACCs is controversial. According to Allolio *et al*., stage IV ACC is not amenable to surgery and mitotane remains the first-line therapy.[9] Conversely, Icard *et al*. reported that debulking surgery along with mitotane in stage IV patients prolongs survival.[10]

The reported median survival of stage IV ACC is less than 12 months. However, there are case reports showing longer survival in patients with stage IV disease, with surgery and chemotherapy.[11] Various clinical trials showing varying effects of different chemotherapy agents like mitotane, EDP (Etoposide, doxorubicin and cisplatin) and sunitinib have shown prolonged survivals in individual stage III/IV patients.[12,13]

Prognosis depends largely on tumor stage. In a study conducted by Icard P *et al*. of 253 patients, the overall survival rate was 38% and the 5-year survival rates were as follows: for stage I 60%; stage II 58%; stage III 24% and stage IV 0%. The overall 5-year survival in different series ranged between 16 and 38%. Median survival for metastatic disease (stage IV) at the time of diagnosis is still consistently less than 12 months.[9,10] The average survival time for untreated patients is 2.5 months.[14] In our case, the patient with stage IV disease survived for 2 months after diagnosis without treatment.

In summary, nonfunctioning ACC is a very rare childhood tumor. Its early detection and appropriate treatment remains a continuing challenge. Although neuroblastoma is the commonest intra-abdominal malignant childhood tumor, in cases with a large nonfunctioning adrenal lesion with pulmonary metastasis and no definite evidence of bone metastasis, a nonfunctioning ACC, though very rare in children, should be kept in differential diagnosis.
